# Autologous stem cell transplant for refractory and relapsed peripheral T-cell lymphoma: a retrospective study in China

**DOI:** 10.7150/jca.89404

**Published:** 2024-01-01

**Authors:** Chen Zhang, Jie Lv, Jili Deng, Weiping Liu, Xiaopei Wang, Yuqin Song, Jun Zhu

**Affiliations:** Key Laboratory of Carcinogenesis and Translational Research (Ministry of Education), Department of Lymphoma, Peking University Cancer Hospital & Institute, Beijing, China.

## Abstract

**Objective** To evaluate the efficacy and prognostic factors of high-dose therapy/autologous stem cell transplantation (HDT/ASCT) in treating refractory and relapsed peripheral T-cell lymphoma (R/R PTCL).

**Methods** We included medical records from 48 R/R PTCL patients treated with HDT/ASCT at the Beijing Cancer Hospital from January 2003 to December 2021, and these patients were followed up.

**Results** We followed up with patients for a median of 71.0 months (interquartile range 48.8-124.4 months). The progression-free survival (PFS) at five years was 43.4%, and the five-year overall survival (OS) was 54.7. The five-year PFS and subgroups were as follows: 14 patients with anaplastic large-cell lymphoma (57.1%, 62.9%), 14 patients with NK/T-cell lymphoma (NKTCL) (28.6%, 28.6%), nine with angioimmunoblastic T-cell lymphoma (44.4%, 51.9%), and 11 with PTCL not otherwise specified (41.6%, 80.8%). Univariate analysis revealed that females had a better PFS than males (hazard ratio [HR] = 0.301, 95% confidence interval [CI] 0.091-0.996, P = 0.049); the NKTCL type had worse OS than the non-NKTCL type (HR = 0.292, 95% CI 0.122-0.698, P = 0.006); the patients with the relapsed disease did better than those with refractory disease (HR for PFS: 0.161, 95% CI 0.072-0.357, P < 0.001; HR for OS: 0.171, 95% CI 0.066-0.444, P < 0.001). The PIT score was significantly better for T-cell lymphoma with score = 0 than for score ≥ 1 group (HR for PFS: 0.261, 95% CI 0.109-0.625, P = 0.003; HR for OS: 0.305, 95% CI 0.111-0.842, P = 0.022). The pre-transplantation disease status also influences survival. Patients who achieved complete response (CR) did better (HR for PFS: 0.104, 95% CI 0.044-0.247, P < 0.001; HR for OS: 0.139, 95% CI 0.050-0.383, P < 0.001). Pre-transplantation status was an independent influencing factor associated with PFS and OS (better survival in those achieving CR) (HR for PFS: 0.126, 95% CI 0.030-0.530, P = 0.005; HR for OS: 0.154, 95% CI 0.040-0.603, P = 0.007); the pathological classification independently influenced OS (better in the those with non-NKTCL) (HR = 0.210, 95% CI 0.081-0.549, P = 0.001). CR, with a PIT score of 0 (n = 17), was associated with more prolonged PFS. None of the 48 patients experienced HDT/ASCT-related deaths.

**Conclusion** HDT/ASCT as a salvage therapy for R/R PTCL patients can partially improve outcomes with a favorable safety profile. Prospective, randomized, and controlled studies are necessary to validate the value of HDT/ASCT for patients with diverse pathological subtypes and pre-transplantation states.

## Introduction

Peripheral T-cell lymphoma (PTCL) is a heterogeneous disease. It has a high incidence in Asian countries, accounting for approximately 20-30% of all lymphomas. PTCL includes peripheral T-cell lymphoma not otherwise specified (PTCL-NOS), angioimmunoblastic T-cell lymphoma (AITL), systemic anaplastic large-cell lymphoma (ALCL), and NK/TCL 1-2. The CHOP-based regimen is the first-line therapy 3-4. Except for anaplastic lymphoma kinase (ALK)-positive ALCL, patients with PTCL suffer poor outcomes 5. Given its high recurrence rate, HDT/ASCT is recommended as a consolidation therapy for patients with most subtypes after the initial response. Although new medications frequently become available 6-12, the efficacy of salvage therapy remains unsatisfactory for patients with refractory/relapsed (R/R) diseases; drug resistance and rapid disease progression are common. Therefore, effective treatments are sought to improve outcomes. In our study, we summarized the efficacy of HDT/ASCT and factors influencing outcomes in patients with R/R disease.

## Cases and methods

### Clinical data

Patients with R/R PTCL receiving HDT/ASCT at the Beijing Cancer Hospital from January 2003 to December 2021 were included. All patients signed informed consent forms for treatment.

A retrospective analysis was performed on 48 R/R PTCL patients receiving HDT/ASCT in the Beijing Cancer Hospital from January 2003 to December 2021. All patients met the diagnostic criteria for R/R PTCL. We recorded medical data and followed up with patients. The median age was 33 years (range 14-66), including 38 males and ten females. Pathological subtypes were as follows: 14 patients with ALCL, 14 with NKTCL, nine with AITL, and 11 with PTCL-NOS. Patients with relapsed disease accounted for 60.4% (29/48), and patients with refractory disease accounted for 39.6% (19/48); ten had local diseases, and the remaining 38 were at stages III to IV; 56.3% experienced B symptoms at onset (e.g., fever, night sweats, and weight loss), and 41.7% suffered elevated lactate dehydrogenase (≥ 240 U/L); only seven patients had bone marrow involvement at diagnosis; 27 had a PIT score of ≥ 1. The prior first-line chemotherapy regimens included CHOP, CHOPE, alternating CHOPE/ GDP, and CHOP-L (only in NK/TCL). There were six cycles (median; range = two to eight cycles); the salvage chemotherapies were DICE, GDP, and GEMOX, with a median of four cycles (range of two to five cycles). The pre-transplantation status was CR in 28 patients, partial response (PR) in 11, stable disease (SD) in three, and progressive disease (PD) in six (Table 1). All patients were evaluated for organ function and comorbidities before transplantation, except for severe infections. All others had clinical indications for autologous stem cell transplantation.

### Stem cell collection and transplantation

Stem cell mobilization was performed after two to three cycles of salvage therapy, and the conditions were confirmed by bone marrow biopsy prior to mobilization. All patients received 10 μg/Kg G-CSF once daily in three mobilization regimens, including mobilization with chemotherapy, disease-specific chemotherapy, or mobilization without chemotherapy (“steady-state”). Peripheral blood samples were collected for routine hematology and CD34 cell count (starting after 2014) after three consecutive days of injection, and the timing of stem cell collection was determined according to test results. A median count of 3.79 ×10^6^/Kg (1.22-11.18 × 10^8^/Kg) mononuclear cells and 2.13 × 10^6^/Kg (0.833-11.19 × 10^6^/Kg) CD34 cells were collected. Only two patients received a combination with plerixafor in mobilization. The stem cells were cryopreserved at -80 °C. Preconditioning regimens included BEAM (carmustine 300 mg/m^2^/d, Day -7; etoposide 100 mg/m^2^/q12h, Day -6 to -3; cytarabine 100 mg/m^2^/q12h, Day -6 to -3; melphalan 140 mg/m^2^/d, Day -2), CBV (cyclophosphamide 1250 mg/m^2^/d, Day -5 to -2; carmustine 300 mg/m^2^/d, Day -6; etoposide 200 mg/m^2^/d, day -5 to -2) and total body irradiation (TBI) with high-dose CTX (TBI total dose 12 Gy, Day -6 to -4; cyclophosphamide 60 mg/kg, Day -3 to -2). The median time of white blood cell implantation was ten days. The median interval to platelet implantation was 12 days. Three patients developed hematogenous infections during transplantation, including one with infectious toxic shock, two with Grade 2 liver impairment, one with interstitial pneumonia, and one with dysbacteriosis-related diarrhea. There was no transplantation-related death.

### Efficacy evaluation and follow-up

During treatment, all patients were evaluated for efficacy every two cycles, pre-transplantation, and 6-8 cycles post-transplantation. We followed patients every 3 to 6 months for 2 years and every 6 to 12 months until disease progression or death. Clinical outcomes were assessed according to the Lugano Lymphoma Response Criteria. The Deauville scoring system would be applied if PET-CT was used for the efficacy evaluation. Follow-ups were achieved through outpatient visits, hospitalizations, and phone calls. OS was measured from the start of conditioning chemotherapy to death or final follow-up. Progression-free survival (PFS) is from the start of conditioning chemotherapy to tumor progression or final follow-up.

### Statistical method

The statistical analysis was based on the software SPSS 27.0 and Graphpad Prism 7.0. The survival analysis used the Kaplan-Meier method with the survival curve plotted. The log-rank test was used to compare the survivals among groups. P < 0.05 defined significance. We used log-rank tests and Cox regression models to assess univariate and multivariate impacts. To identify prognostic variables for PFS and OS, we performed univariate analysis for sex, age at transplantation, number of extranodal involvement, stage of disease, B symptoms, PIT score, the level of lactate dehydrogenase before transplantation, disease status before transplantation, time to neutrophil engraftment, time to platelet engraftment, CD34^+^ cell infusion dose, conditioning regimen, and R/R stage.

## Results

### Efficacy evaluation

The efficacy evaluations were completed within 8 weeks after the HDT/ASCT. There was CR in 30 patients, PR in six, SD in four, and PD in eight. Of the 28 who achieved CR before transplantation, nine progressed after transplantation, and five died due to disease progression. Of the 11 who achieved PR, 11 progressed, and eight died. Three patients achieved SD, all of whom progressed and died.

### Survival analysis

**Figure 1:**
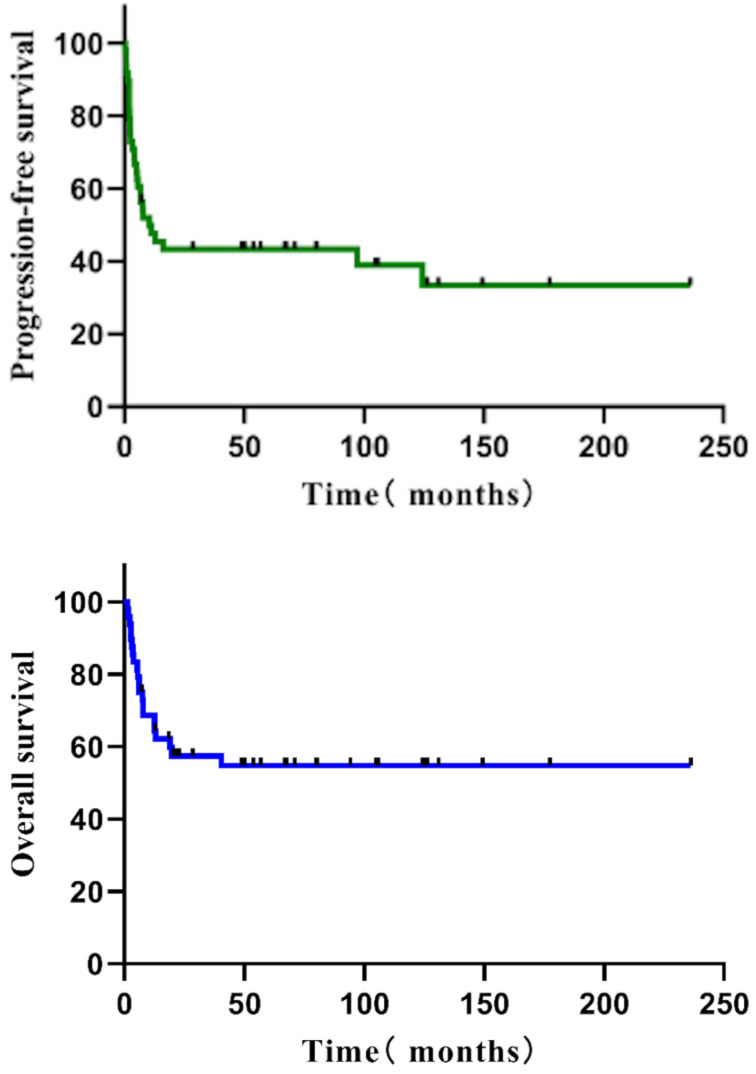
The PFS and OS of all R/R PTCL

**Figure 2:**
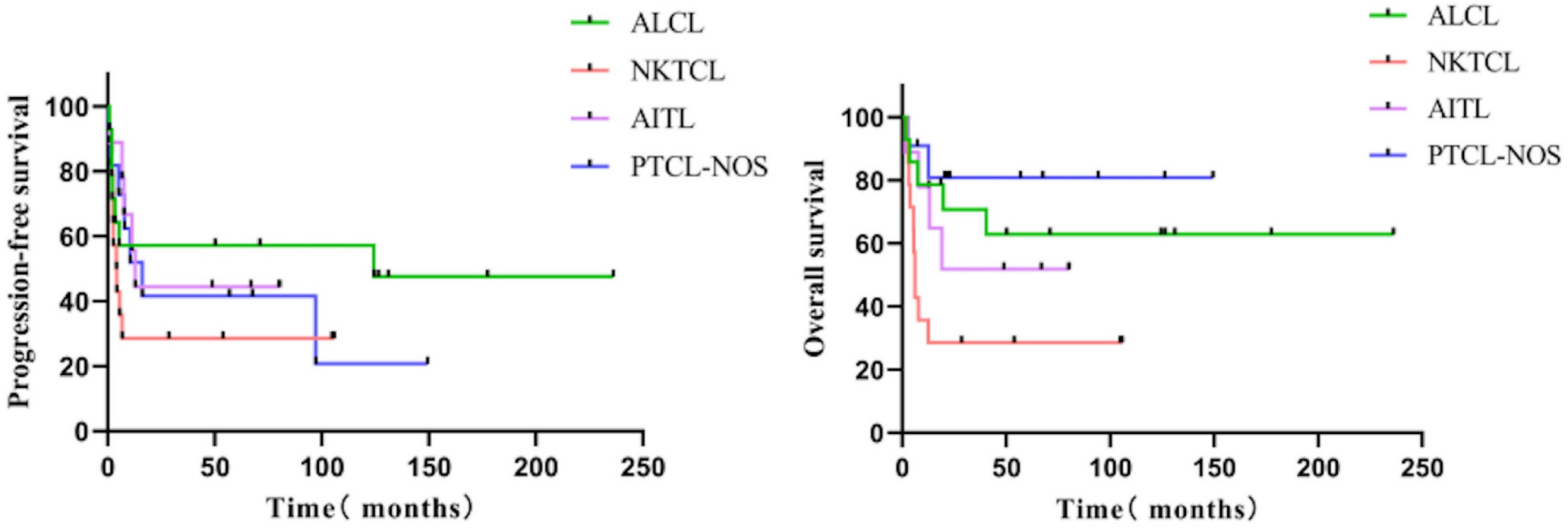
The PFS and OS of various pathological subgroups

Follow-up was 71.0 months (median; interquartile range = 48.8-124.4 months). The five-year PFS of all 48 individuals with R/R PTCL was 43.3 %, and the five-year OS was 54.7% (Figure 1). The five-year PFS and OS of various subgroups were as follows: 14 patients with ALCL (57.1%, 62.9%), 14 patients with NK/TCL (28.6%, 28.6%), nine with AITL (44.4%, 51.9%), and 11 with PTCL-NOS (41.6%, 80.8%) (Figure 2). None of the 48 patients experienced HDT/ASCT-related deaths.

### Analysis of factors influencing outcomes

The univariate analysis revealed that the females had a better PFS than the males (hazard ratio [HR] = 0.301, 95% confidence interval [CI] 0.091-0.996, P = 0.049) (five-year PFS 70.0% vs. 36.2%); the non-NKTCL type had a better OS than those of the NKTCL type (HR = 0.292, 95% CI 0.122-0.698, P = 0.006) (five-year OS 65.5% vs. 28.6%). The patients with relapsed disease had better outcomes than those with refractory disease (HR for PFS: 0.161, 95% CI 0.072-0.357, P < 0.001; HR for OS: 0.171, 95% CI 0.066-0.444, P < 0.001). Five-year PFS was 64.9% vs. 10.5%, and five-year OS was 79.0% vs. 19.7%. Survival was significantly better with PIT score = 0 than with PIT score ≥ 1 (HR for PFS: 0.261, 95% CI 0.109-0.625, P = 0.003; HR for OS: 0.305, 95% CI 0.111-0.842, P = 0.022). Five-year PFS was 71.4% vs. 20.4%, and five-year OS was 76.2% vs. 35.9%). The pre-transplantation disease status also influenced survival. Patients with CR did better (HR for PFS: 0.104, 95% CI 0.044-0.247, P < 0.001; HR for OS 0.139, 95% CI 0.050-0.383, P < 0.001). The five-year PFS was 71.1% vs. 5.0%, and the five-year OS was 81.8% vs. 25.0%) (Table 2).

The multivariate analysis showed that pre-transplantation status was independently associated with PFS (better survival in those with CR) (HR for PFS: 0.126, 95% CI 0.030-0.530, P = 0.005); pre-transplantation status (better survival in CR patients) (HR = 0.154, 95% CI 0.040-0.603, P = 0.007), and pathological classification were independent influencing factors for OS (better survival in the patients with non-NKTCL) (HR = 0.210, 95% CI 0.081-0.549, P = 0.001) (Table 2).

OS was prolonged in patients with CR (HR for PFS: 0.126, 95% CI 0.030-0.530, P = 0.005; HR for OS: 0.154, 95% CI 0.040-0.603, P = 0.007). The pathological classification was an independent influencing factor for OS (better survival in the non-NKTCL group) (HR = 0.210, 95% CI 0.081-0.549, P = 0.001) (Table 2).

**Figure 3:**
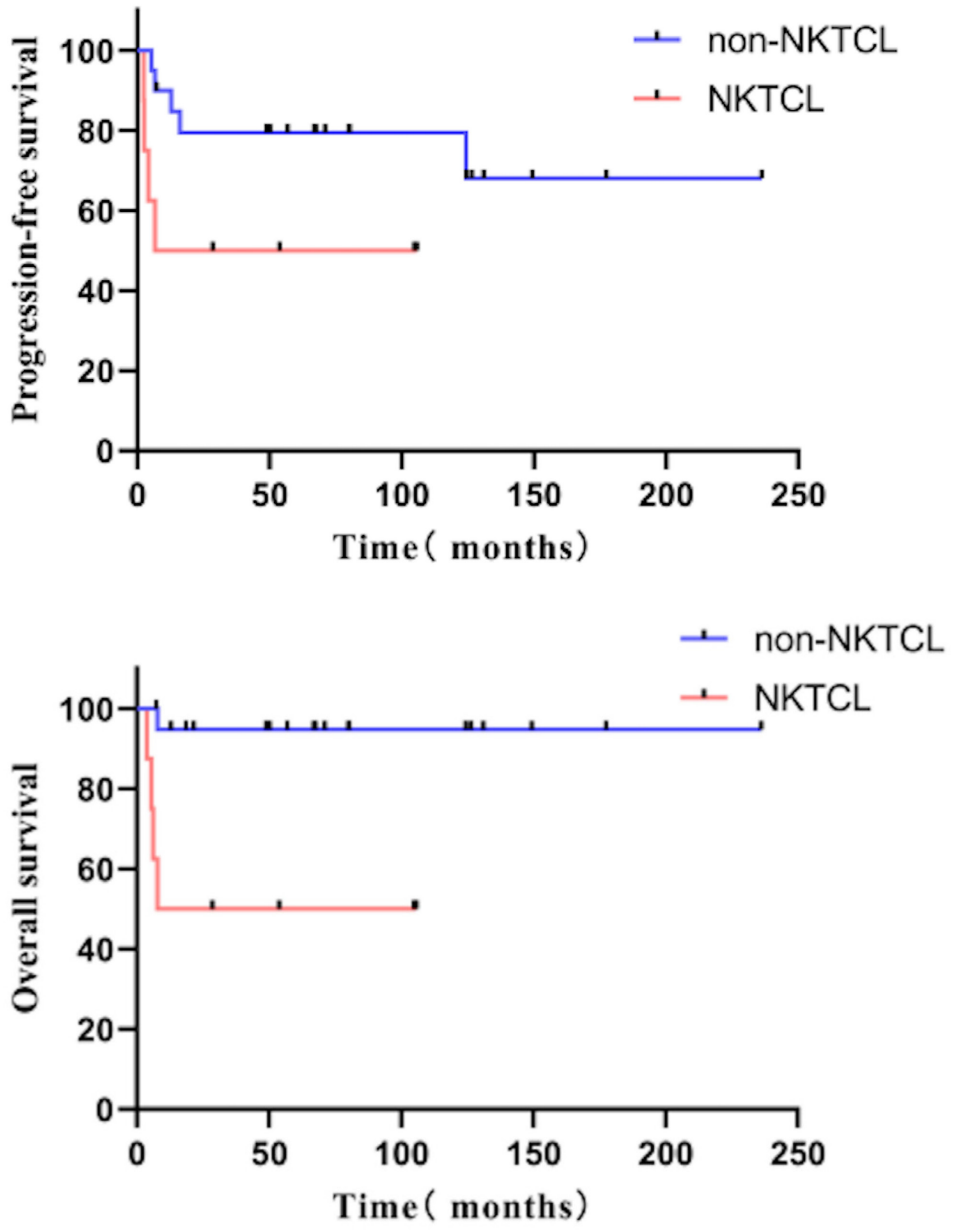
The PFS and OS of patients with NKTCL and non-NKTCL pathological type

In the subgroup analysis, 28 patients achieved CR before transplantation with a five-year PFS of 71.1% and a five-year OS of 81.8%. Patients achieving CR with a PIT score of 0 (n = 17) had more prolonged PFS (p = 0.047, HR = 4.212 [1.016-17.451]) than those with PIT ≥ 1 (n = 11), and five-year PFS was 88.2% vs. 42.4%. Patients achieving CR before transplantation and pathological type non-NKTCL (n = 20) had a more significant benefit from transplantation than those with NKTCL (n = 8), with five-year PFS 79.4% vs. 50.0% (p = 0.078) and five-year OS 94.7% vs. 50.0% (p = 0.020) (Figure 3).

## Discussion

PTCL refers to a large class of heterogeneous diseases, with most subtypes having poor outcomes and an overall five-year survival of only about 30% 13. High-dose chemotherapy with autologous hematopoietic stem cell transplantation is the first-line consolidation treatment for relapsed/refractory patients. For treatment-naïve patients, few large-scale prospective randomized controlled studies have been conducted to investigate this regimen; however, several extensive sample-size retrospective studies suggested that consolidation transplantation prolonged survival 14-15. Nevertheless, about one-third of the patients experienced rapid progression before transplantation. Some studies explored the suitable timing for transplantation and found that the outcome of consolidating transplantation after reaching the initial response was better than that of salvage transplantation in patients with relapse 16. Consolidation via first-line stem cell transplantation might improve survival; nevertheless, relapse or disease progression occurs in many patients, and there are no effective treatments for patients with relapsed or refractory disease. The recommendation is to consider a clinical trial. A study of 153 patients with R/R PTCL receiving chemotherapy alone but no subsequent transplantation showed a median PFS of only 5 months and a median OS of only 13.7 months 17. Studies on some agents such as pralatrexate, brentuximab, vedotin (a CD30-targeting antibody-drug conjugate), and HDAC inhibitors have shown efficacy in patients with R/R disease but no substantial improvement in survival 9, 18-19. The response rates are low for most drugs, and the response duration is unsatisfactory.

Given the high relapse rates in PTCL, NCCN guidelines recommend ASCT as an option for consolidation in patients with most the subtypes of PTCL 20. However, there are less relevant reports on the role of ASCT in relapsed and refractory disease. There are no randomized trials evaluating the benefit of this group of patients. The Memorial Sloan-Kettering Cancer Center compared ASCT for chemosensitive R/R PTCL (n=24) with R/R diffuse large B cell lymphoma (DLBCL) (n=86). The 5-year PFS (24% vs. 34%, P=0.14) and OS (33% vs. 39%, P=0.64) were similar between PTCL and DLBCL. In this study, age-adjusted IPI predicted PFS and OS in multivariate analysis. At the same time, some other reports demonstrated that salvage transplantation helped these kinds of patients achieve five-year OS of 34% to 48% 21-23, suggesting a significant survival benefit compared with those receiving chemotherapy alone. The treatment of primary refractory diseases is more difficult. A study from the University of Michigan Health System for nearly 30 years, which included 93 patients with primary refractory PTCL, confirmed that even for this group of patients, those sensitive to salvage chemotherapy still had survival benefits from stem cell transplantation 24.

In relapsed patients, ASCT can salvage about a third of chemosensitive ones, with the best outcomes for ALCL 25. For relapsed/refractory NKTCL, an allogeneic stem cell transplant can be considered and is recommended in eligible patients. Our study, concluded that non-NKTCL type patients have better survival, so for NKTCL, we suggest receiving allogeneic transplantation directly. However, we did not see a significant survival difference between ALCL and PTCL, not otherwise specified type, due to the small number of cases.

Allogeneic stem cell transplantation (Allo-SCT) may be considered for PTCL patients with multiple relapses or failure of autotransplantation. The studies by CIBMTR compared autograft and allograft transplantations and found similar long-term survival results but lower NRM in the former 26. A large retrospective study evaluated 76 patients with R/R PTCL who had undergone ASCT or Allo-SCT. Among them, 41 underwent ASCT. The four year PFS and OS rates were 38% and 50%. The outcomes were superior for patients who had attained CR before ASCT. The decision for ASCT should be depended on the efficacy of salvage therapy. The above findings were similar to the conclusion of our study. In the other 35 patients who had undergone allo-SCT, the four year PFS and OS were 25% and 36%, respectively. The non-relapse mortality was higher in allo-SCT than in ASCT. In addition, an extensive systematic review and meta-analysis included 1765 patients from 30 studies (880 patients who underwent allo-SCT and 885 who underwent ASCT). In the ASCT group, a 5-year PFS and OS were 40% and 53%, similar to our study. This study concludes that PFS and OS were similar in the allo-SCT and ASCT groups. However, in allo-HCT, the cost, donor availability, and high risk of complications limited its application 27. Due to the large number of pathological subtypes of PTCL and the lack of large-scale randomized controlled studies, treatment for these patients with a relapsed or refractory disease is a substantial challenge 28.

In conclusion, we found that HDT/ASCT is safe and effective for patients with R/R PTCL; it can improve survival in PTCL patients with non-NKTCL pathologic type and chemotherapy sensitivity, especially those achieving CR before transplantation. Extensive prospective randomized controlled studies in patients with various pathological subtypes/different response states must confirm the survival benefit of high-dose chemotherapy with autologous transplantation of hematopoietic stem cells.

## Figures and Tables

**Table 1:** The clinical characteristics of the 48 patients with R/R PTCL

		N (%)
Sex	Male	38 (79.2%)
	Female	10 (20.8%)
Age	33 (14-66)	
Pathological subtypes	ALCL	14 (29.2%)
	NKTCL	14 (29.2%)
	AITL	9 (18.8%)
	PTCL-NOS	11 (22.9%)
Refractory or recurrence	Recurrence	29 (60.4%)
	Refractory	19 (39.6%)
Stage	I-II	10 (20.8%)
	III-IV	38 (79.2%)
B symptoms	Yes	27 (56.3%)
	No	21 (43.7%)
Elevated LDH	Yes	20 (41.7%)
	No	28 (58.3%)
Bone marrow invasion	Yes	7 (14.6%)
	No	41 (85.4%)
PIT score	0	21 (43.8%)
	≥ 1	27 (56.2%)
Pre-transplantation status	CR	28 (58.3%)
	PR	11 (22.9%)
	SD	3 (6.3%)
	PD	6 (12.5%)
Preconditioning scheme	BEAM	23 (47.9%)
	CBV	20 (41.7%)
	TBI	5 (10.4%)

**Table 2:** Univariate and multivariate Cox regression analysis for PFS and OS

		PFS				OS			
		Univariate Cox regression analysis		Multivariate Cox regression analysis		Univariate Cox regression analysis		Multivariate Cox regression analysis	
		HR (95% CI)	P	HR (95% CI)	P	HR (95% CI)	P	HR (95% CI)	P
Female		0.301 (0.091-0.996)	**0.049**	0.335(0.088-1.284)	0.111	0.299 (0.069-1.286)	0.105		
Age at transplantation ≥ 40 years old		1.197 (0.566-2.530)	0.638			1.689 (0.715-3.988)	0.232		
External lymph node involvement ≥ 2		0.826 (0.389-1.753)	0.618			0.670 (0.270-1.662)	0.388		
Stage III-IV		0.659 (0.279-1.555)	0.341			0.600 (0.232-1.547)	0.290		
Non-NKTCL		0.554 (0.253-1.210)	0.138			0.292 (0.122-0.698)	**0.006**	0.210 (0.081-0.549)	**0.001**
B symptoms		1.175 (0.559-2.468)	0.671			1.389 (0.590-3.273)	0.452		
Elevated LDH		2.059 (0.973-4.357)	0.059			1.546 (0.637-3.755)	0.336		
PIT score = 0		0.261 (0.109-0.625)	**0.003**	0.790(0.254-2.459)	0.685	0.305 (0.111-0.842)	**0.022**	1.000 (0.317-3.150)	1.000
Pre-transplantation disease status: CR		0.104 (0.044-0.247)	**<0.001**	0.126(0.030-0.530)	**0.005**	0.139 (0.050-0.383)	**<0.001**	0.154 (0.040-0.603)	**0.007**
Neutrophil implantation ≤ 10 days		1.235 (0.588-2.592)	0.577			1.828 (0.738-4.532)	0.193		
PLT implantation ≤ 12 days		1.308 (0.613-2.789)	0.488			1.511 (0.624-3.655)	0.360		
CD34+ cell infusion dose ≥ 2.0× 10^6^/Kg		0.753 (0.363-1.562)	0.446			1.028 (0.433-2.442)	0.950		
Conditioning regimen	TBI (n = 4)	Ref							
	BEAM (n = 23)	1.536 (0.351-6.724)	0.569			0.842 (0.182-3.902)	0.826		
	CBV (n = 21)	1.093 (0.242-4.949)	0.908			0.936 (0.204-4.283)	0.932		
Recurrence		0.161 (0.072-0.357)	**<0.001**	0.959 (0.246-3.747)	0.952	0.171 (0.066-0.444)	**<0.001**	0.597 (0.172-2.066)	0.415
